# Peptidomimetic inhibitors of L-plastin reduce the resorptive activity of osteoclast but not the bone forming activity of osteoblasts *in vitro*

**DOI:** 10.1371/journal.pone.0204209

**Published:** 2018-09-24

**Authors:** Meenakshi A. Chellaiah, Sunipa Majumdar, Hanan Aljohani

**Affiliations:** Department of Oncology and Diagnostic Sciences, School of Dentistry, University of Maryland, Baltimore, Maryland, United States of America; Charles P. Darby Children's Research Institute, 173 Ashley Avenue, Charleston, SC 29425, USA, UNITED STATES

## Abstract

Sealing ring formation is a requirement for osteoclast function. We have recently identified the role of an actin-bundling protein L-plastin in the assembly of nascent sealing zones (NSZs) at the early phase of sealing ring formation in osteoclasts. TNF-α signaling regulates this actin assembly by the phosphorylation of L-plastin on serine -5 and -7 residues at the amino-terminal end. These NSZs function as a core for integrin localization and coordinating integrin signaling required for maturation into fully functional sealing rings. Our goal is to elucidate the essential function of L-plastin phosphorylation in actin bundling, a process required for NSZs formation. The present study was undertaken to determine whether targeting serine phosphorylation of cellular L-plastin would be the appropriate approach to attenuate the formation of NSZs. Our approach is to use TAT-fused small molecular weight amino-terminal L-plastin peptides (10 amino acids) containing phospho- Ser-5 and Ser-7. We used peptides unsubstituted (P1) and substituted (P2- P4) at serine-to-alanine residues. Immunoblotting, actin staining, and dentine resorption analyses were done to determine cellular L-plastin phosphorylation, NSZ or sealing ring formation, and osteoclast function, respectively. Immunoblotting for bone formation markers, Alizarin red staining and alkaline phosphatase activity assay have been done to determine the effect of peptides on the mineralization process mediated by osteoblasts. Transduction of unsubstituted (P1) and substituted peptides at either Serine 5 or Serine 7 with Alanine (P3 and P4) demonstrated variable inhibitory effects on the phosphorylation of cellular L-plastin protein. Peptide P1 reduces the following processes substantially: 1) cellular L-plastin phosphorylation; 2) formation of nascent sealing zones and sealing rings; 3) bone resorption. Substitution of both Serine-5 and -7 with Alanine (P2) had no effects on the inhibitory activities described above. Furthermore, either the L-plastin (P1-P5) or (P6) control peptides had a little or no impact on the a) assembly/disassembly of podosomes and migration of osteoclasts; b) mineralization process mediated by osteoblasts in vitro. Small molecular weight peptidomimetics of L-plastin inhibits bone resorption by osteoclasts via attenuation of NSZ and sealing ring formation but not bone formation by osteoblasts in vitro. The L-plastin may be a valuable therapeutic target to treat and prevent diseases associated with bone loss without affecting bone formation.

## Introduction

Osteoclasts (OCs) exist in two functional states: a) the migratory state and b) the resorptive state. OCs show different types of cell-matrix contacts in these states. a) Migratory osteoclasts attach and move over the bone matrix via the formation of F-actin enriched podosomes [1,2]. b) The adhesion of osteoclasts to the bone during bone resorption leads to the formation of a sealing ring, an actin-rich ring-like adhesion zone circumscribing an area of bone resorption. The process of sealing ring formation has been considered to be a marker of osteoclast activation for bone resorption. Many questions about the actin remodeling processes involved in the formation of sealing rings remain to be answered.

Sealing rings consisting of stable actin filaments which generate tight sealing zones on the bone surface during bone resorption by osteoclasts. A major reorganization of the actin filament is required during the formation of the sealing ring. We have previously demonstrated the formation of actin aggregates at the early stage of bone resorption in the presence of TNF-alpha (α) or RANKL independent of integrin signaling. These actin aggregates are denoted as nascent sealing zones (NSZs). We proposed NSZs as precursor zones for sealing rings. Lysates made from osteoclasts treated with native mice bone particles (60–80γm size) and TNF- α or RANKL revealed a possible mechanistic role for an actin-bundling protein L-plastin (LPL) in the organization of NSZs [3].

Plastins are a family of three tissue-specific actin-binding proteins (ABPs). Although three isoforms of plastins (L-, T-, and I-plastin) have been characterized, only L and T-plastin have been shown to be involved in cytoskeletal reorganization in signal transduction pathways [4]. T-plastin is expressed in cells from solid tissue, whereas L-plastin (LPL) occurs predominantly in hematopoietic cells. The third isoform, I-plastin, is specifically expressed in small intestine, colon, and kidney [5]. Among the three plastins (T, L, and I plastin), LPL is capable of distinguishing between actin isoforms as it has been shown to efficiently bundle β-actin but not α or γ actin isoforms [6].

Plastins contain an amino-terminal (N-terminal) domain containing two phosphoserine residues (Ser-5 and Ser-7) and Ca2+binding sites flanked by EF-hand motifs followed by two repeated actin-binding domains (ABDs) and calponin homology domain (CH) at the carboxyl-terminal (C-terminal) end. The spatially close ABDs (120Å) of plastins enable them to organize actin filaments into tight bundles [7,8]. LPL localizes to actin-rich membrane structures involved in locomotion, adhesion, and membrane extensions (e.g. filopodia, lamellipodia), [9–11]. LPL is also known as Plastin 2, cytoskeletal associated protein (CAP) or fimbrin.

LPL is, so far, the only of the three plastin isoforms that is phosphorylated in cells [4]. Phosphorylation of LPL occurs on Ser-5 and -7 amino acid upstream of cytoskeleton rearrangements that underlie processes such as chemotaxis and adhesion [10,12,13]. Phosphorylation of L-plastin on residues Ser-5 and -7 occurs in hematopoietic cells [5, 14], but most likely on Ser- 5 exclusively in non-hematopoietic cells [4, 15]. The physiological function of Ser- 7 phosphorylation is not known. LPL phosphorylation stabilizes actin filaments and protect them against depolymerization [16].

We have previously shown cyclical changes in protein and phosphorylation levels of LPL correspond with the time-dependent changes in the organization of actin filaments in osteoclasts subjected to bone resorption. LPL serine phosphorylation-dependent actin bundling promoted NSZs formation at the early phase of sealing ring formation [3]. Our hypothesis here is that the inhibition of phosphorylation of L-plastin has the potential to attenuate sealing ring formation and bone resorption. Studies with TAT-fused LPL peptide containing serine phosphorylation sites in polymorphonuclear neutrophils (PMNs) [9] demonstrates the potential for this peptide transduction to be applied to studies in the osteoclast system.

In this paper, we have investigated the functional role of the phosphorylation region of LPL on NSZs formation. We used TAT-fused small molecular weight (10aa) amino-terminal -LPL peptides ((sNT)-LPL; “1MARG**S**V**S**DEE10”) containing phospho- Ser-5 and Ser-7 (P1; also represented as unsubstituted) and substituted peptides with Ala-5 and -7 for Ser-5 and Ser-7 (P2-P4). We show here the ability of TAT-fused peptides to enter into osteoclasts and suppress the phosphorylation of endogenous LPL competitively and hence NSZs formation and bone resorption. The inhibition was substantial with the P1 peptide as compared with the peptides having a substitution at either Ser-5 or Ser-7 with Ala-5 or Ala-7 (P3 and P4). These observations justify testing the effect in vivo in future studies. Understanding the mechanism in vivo in mice can further direct drug development for this pathway.

## Materials and methods

### Materials

Antibody to L-plastin (SC-16657; Goat) was bought from Santa Cruz Biotechnology, Inc. (Santa Cruz, CA). Antibodies to GAPDH was purchased from R & D Systems (Minneapolis, MN) and Sigma (St. Louis, MO). Protein estimation reagent, molecular weight standards for proteins, and PAGE reagents were bought from Bio-Rad. Cy2- and Cy3-conjugated secondary antibodies were purchased from Jackson Immunoresearch (West Grove, PA). HRP-conjugated secondary antibodies for immunoblotting were obtained from GE Healthcare. Antibody to phosphoserine (p-Serine) was bought from Zymed laboratories (61–8100) or Millipore (AB1603). Alizarin red solution was bought from Life-line Cell Technology (CM-0058; Fredrick, MD) Rhodamine-phalloidin and other chemicals were purchased from Sigma (St. Louis, MO).

### Studies in osteoclasts

#### Mice

C57/BL6 mice (six to eight-week-old mice) were used for osteoclast preparation. These mice were either purchased from Harlan Laboratories or generated in the animal facility of the University of Maryland Dental School. Breeding and maintenance were carried out as per the guidelines and approval of the institutional animal care and use committee (IACUC). Mice were euthanized as per approved protocols (Protocol number 04170006) by the IACUC of the University of Maryland, Dental School, where mice were housed and bred.

#### Preparation of Osteoclast precursors from mice and treatment of osteoclasts with bone particles

C57/BL6 mice were used for osteoclast preparation as described previously [17, 18]. These mice were either purchased from Harlan Laboratories or generated in the animal facility of the University of Maryland Dental School. Breeding and maintenance were carried out as per the guidelines and approval of the institutional animal care and use committee. Osteoclasts were generated *in vitro* using mouse bone marrow (BM) cells as described [17, 18]. Osteoclasts differentiated from RAW 264.7 cells were also used to corroborate a few of the observations made with osteoclasts derived from mouse BM cells. Osteoclasts from RAW 264.7 cells were generated as described previously [19]. We established conditions that offer >90% population of mature multinucleated osteoclasts from both mouse bone marrow and RAW cells.

#### Treatment of osteoclasts with bone particles

After flushing the marrow cells for osteoclast differentiation, long mouse bones (free of cells inside and muscles outside) were washed extensively with PBS and kept in ethanol until use. Long bones were air-dried in the hood and homogenized by a mini blender. Bone particles were sieved, and bone particles 60–80 μm in size were used for experiments. The multinucleated osteoclasts were seen from day four onward from mouse BM or RAW cells. At this stage, osteoclasts were added with sterile native bone particles (100μg /ml medium) for 3–4 h or 12-14h in the presence of TNF-α (20ng/ml).

#### Transduction of TAT-fused sNT-LPL peptides into osteoclasts

After cells were kept in the serum-free α-MEM medium for two hrs. and LPL peptides of interest were added to a final concentration of 100–150 nM to cells in serum-free media [2]. Osteoclasts transduced with peptides of interest and added with bone particles in the presence of TNF-α for 3–4 or 12–14 h were used for lysate preparations. Cells plated on coverslips and treated with peptides were used for actin staining to detect podosomes. After transduction for 30min., osteoclasts were replated on dentine slices with respective LPL peptide and TNF-α for 3-6h or 12-14h. These cells were used for Actin staining with rhodamine-phalloidin or immunostaining analyses [2,17, 20].

#### Lysate preparation

Following various treatments, osteoclasts were washed three times with cold PBS and lysed in a Triton-containing lysis buffer as described [21]. Cells were rocked on ice for 15 min. and scraped off with a cell scraper. Cell lysates were centrifuged at 15,000 rpm for 5 min., at 4°C, and the supernatant was saved. Protein contents were measured using the Bio-Rad protein assay reagent. Osteoclast cultures were also subjected to a trypan blue dye (Sigma T8154) exclusion test to determine the viability of osteoclasts after various treatments. Cells demonstrated clear cytoplasm with no inclusion of blue dye [3].

#### Immunoprecipitation and immunoblotting (IB) analyses

About 50–100μg of lysate protein from mouse osteoclasts or RAW cell-derived osteoclasts was used for immunoprecipitation and immunoblotting analyses. Immunoprecipitation with an LPL antibody was done as described previously [22]. The proteins were transferred to a PVDF membrane for IB analysis after 10% SDS-PAGE. Blots were blocked with 10% milk in PBS containing 0.5% Tween (PBS-T) for 2–3 h and then incubated with 1:1000 dilutions of a primary antibody of interest for 2–3 h. After three washes for 10 min each with PBS-T, the blot was incubated with a 1:1000 dilution of peroxidase-conjugated species-specific respective secondary antibody for 2h at room temperature. After three washes for 10 min each with PBS-T, protein bands were visualized by chemiluminescence using the ECL kit (Pierce) [22].

#### Immunohistochemistry and actin staining

Osteoclasts were fixed with 3% paraformaldehyde for 20 min and permeabilized with 0.1% Triton X-100 in PBS for 5 min. Subsequently, osteoclasts were stained with a primary (TAT, LPL or integrin αv) and secondary antibody as described [20]. Actin staining was done with rhodamine-phalloidin as described [2,17]. Cells were washed and mounted on a slide in a mounting solution (Vector Laboratories) and sealed with nail polish. Immunostained osteoclasts were photographed with a Bio-Rad and Nikon confocal microscope. Images were stored in TIF image format and processed by Adobe Photoshop (Adobe Systems Inc., Mountain View, CA).

#### Measurement of F-actin content using Rhodamine- Phalloidin binding

Osteoclasts were transduced with LPL and control peptides. For each treatment, four to six wells in 24 well culture dishes were used. Cells were fixed, and rhodamine phalloidin binding to F-actin was done as described [21,22].

#### Dentine resorption lacuna and migration assays

Resorption was evaluated using dentine slices as described previously [2]. After transduction for 30min., osteoclasts were replated on dentine slices for 12-16h to detect resorption lacuna in the presence of respective LPL peptide and TNF-α. Each treatment was done in quadruplicates. Subsequently, dentine slices were stained with Mayer's acid hematoxylin (Sigma) for 6 min followed by washing several times with water. Excess stain in the resorbed area and stained cell debris were removed with a cotton swab. Subsequently, the pits were scanned in a confocal microscopy to determine the extent of pit formation and pit area [17]. Images were stored in TIF format and processed by Adobe Photoshop (Adobe Systems Inc.).

Cell migration (phagokinesis and transwell migration) assays were done as described previously [17]. Osteoclasts transduced with sNT-LPL peptides (P1, P2, P5, and P6) were used for these assays. Three to four transwells were used for each treatment. Migrated cells at the bottom of the wells were counted in 3–4 independent fields/transwell filter. Data are presented as migrated cells/field (mean±SD). The experiment was repeated with three osteoclast preparations. In phagokinesis assay, cell motility was assessed by measuring the areas free of gold particles. By using a grided reticule (Boyce Scientific, Inc.) in the eyepiece of a Nikon microscope, areas free of gold particles were measured using a 10X objective. Areas free of gold particles were represented as area moved in mm^2^ [17].

### Studies in osteoblasts

#### Cell culture

We used both MC3T3 and UMR106 cells for the assays to detect bone mineralization. Approximately 3X10^5^ MC3T3 cells were cultured in osteogenic medium (αMEM + 10% fetal bovine serum supplemented with 50mM ascorbic acid and 10mM β-glycerophosphate) for seven days in the presence of sNT-LPL peptides of interest (100nM). UMR-106 cells were plated at 0.6 x 10^6^ density in a 6-well plate for ALP activity assay and 0. 4 X 10^6^ in 24-well plate for Alizarin Red S staining (ARS). Cells were maintained in Dulbecco's Modification of Eagles Medium (DMEM) containing 10% FBS (Benchmark), 1% penciling/streptomycin, and 0.05% gentamicin. After reaching 90–95% confluency, the culture medium was replaced with osteogenic medium (7 mM β-glycerophosphate (Sigma), 50 μM ascorbic acid 2- phosphate (Sigma)) for seven days [19]. sNT-LPL peptides (100ng) of interest were added to cultures every 20-24h for seven days. We used cell counting Kit-8 (CCK-8; Sigma) in parallel cultures treated as above to determine the viability.

#### Alizarin Red S staining (ARS)

After seven days, cells washed three times with PBS were fixed with 4% formaldehyde in PBS for 30 min at room temperature (RT). After washing one time with PBS, 2% Alizarin red stain solution was added to each well and incubated for 45 min at RT. Then, wells were washed with tap water three times to remove unincorporated excess dye before scanning the plate in an EPSON Perfection V200 Photo scanner. Magnified pictures of the wells were taken using a phase contrast microscopy (Nikon) using 10X or 20X objective.

#### Alkaline phosphatase (ALP) activity assay

For ALP activity, cells were washed with cold PBS three times and added with lysis buffer (50 mM Tris, 0.1% Triton-x, 1 mM MgCl_2_, and 100 mM glycine). Lysates were centrifuged at 10,000X g (13000 RPM) for 5 min. An equal amount of supernatant protein was used triplicates in a 96-well plate to measure the activity. Ρ-Nitrophenyl phosphate (100 μl; Sigma) was added to each well and absorbance was measured at 405 nm using microplate reader (Cytation3 image reader) with software (Gen5 version 2.09).

#### Immunoblotting

UMR-106 cells were grown as described above and the sNT-LPL peptide of interest (100nM) was added to cultures every 20-24h for seven days. Cells grown in osteogenic medium only were used as controls. The cells were lysed in RIPA lysis buffer containing protease inhibitors (2). Lysates were incubated on ice for 15 minutes and then centrifuged at 10000 x g (13,000 RPM) for 15 minutes at 4°C. The supernatant was collected, and the protein concentration was determined using Bradford assay. An equal amount of lysates (10 μg) were subjected to either 8% or 10% SDS-PAGE and transferred to PVDF membranes. Western blotting was done with antibodies to collagen (1:2000) (Novus Biologicals), Runx2 (1:1000) (Santa Cruz), or osterix (1:1000) (Millipore) as described previously [21,22]. Western blotting with a GAPDH antibody (Sigma) was used as a control for loading.

### Statistical analysis

Data obtained represent the response of the osteoclast culture as a whole (>85% multinucleated giant cells and ~10–15% osteoclast precursors). Statistical significance was determined using either analysis of variance ANOVA or student’s t-Test (INSTAT; Version 6.0, Graph Pad software, Graph Pad Inc, San Diego, CA). Results are presented as means ± SD. A probability value< 0.05 was considered to be statistically significant and <0.01 was supposed to be highly significant.

## Results

Previous studies have shown that the actin bundling process is dependent on the phosphorylation of LPL [3, 23–25]. Most experiments concerning LPL function have been performed in PMNs. Jones et al. analyzed the function of LPL by introducing LPL -derived peptides containing the NT-region into PMNs [9]. We have previously characterized the dynamic localization of the proteins L-Plastin and cortactin in regulating actin polymerization. We also defined the possible molecular interactions involved in the process of NSZs and sealing ring formation by these proteins [3]. In this paper, we identify the essential function of phosphorylation on Ser- 5 and 7 by TNF-α signaling in actin bundling, a process required for NSZ formation by LPL.

**Fig 1:**
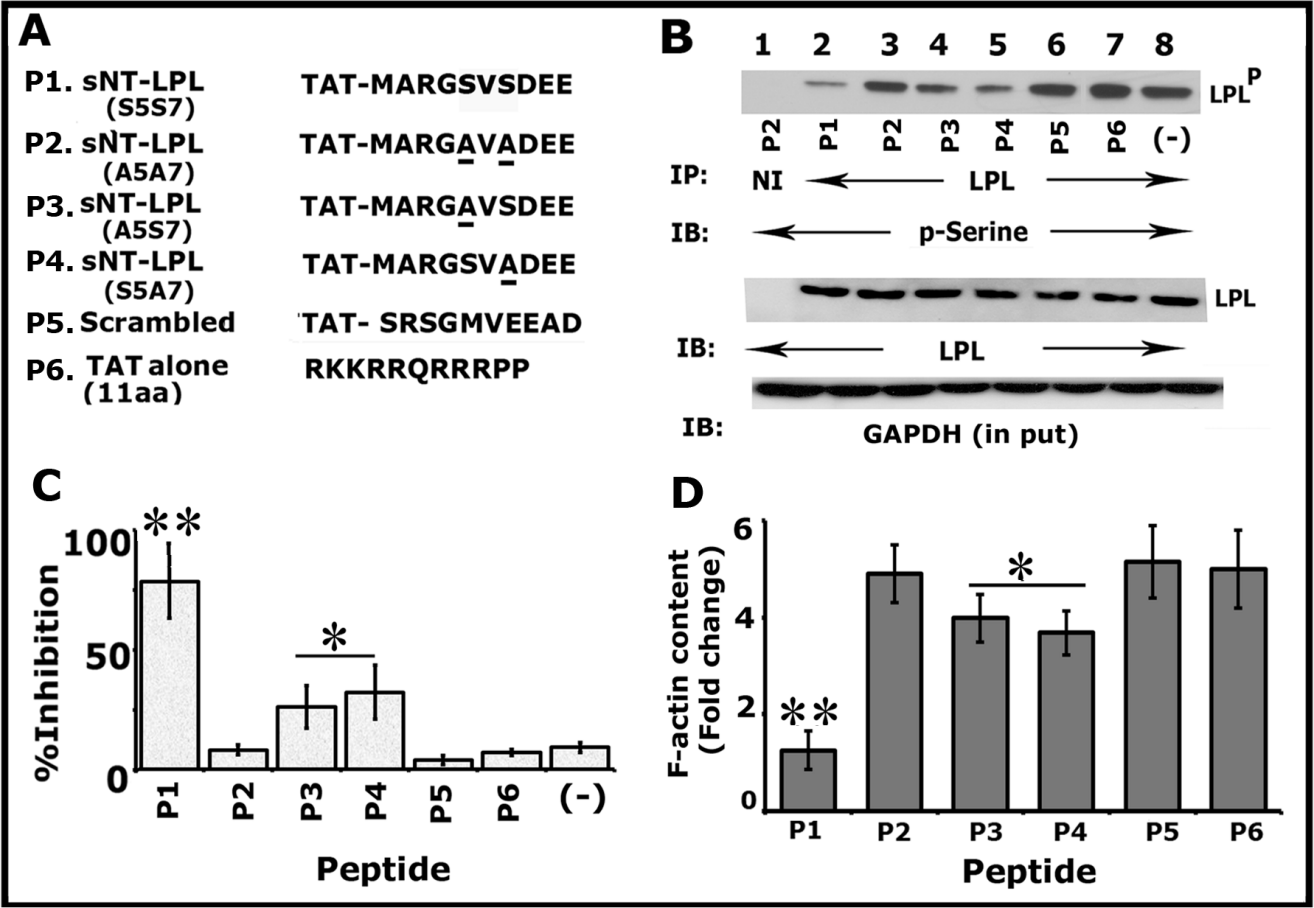
TAT-fused small molecular weight amino-terminal LPL (sNT-LPL) has the potential to reduce the phosphorylation of cellular LPL and total F-actin content. (A) Amino acid sequences of TAT-fused sNT-LPL (10aa) and control TAT alone (11aa) peptides are shown: P1) unsubstituted (S5S7); P2) double substituted (S5S7 to A5A7); P3) Ser-5 substituted with Ala (A5S7); P4) Ser-7 substituted with Ala (S5A7); P5) scrambled; P6) control TAT alone peptide. (B and C) Immunoprecipitation and immunoblotting analyses: Equal amount of osteoclast lysates were immunoprecipitated with an antibody to LPL and subjected to immunoblotting (IB) with a p-Serine antibody (top). This blot was stripped and blotted with an LPL antibody (Panel B; middle). An equal amount of total protein (Input) used for immunoprecipitation was assessed by direct immunoblotting of lysates with a GAPDH antibody. These results represent one of the three experiments performed with the similar results. Percent inhibition of phosphorylation of cellular LPL with indicated peptides is provided as a graph. **p<0.01; *p<0.05 versus double substituted (A5A7), scrambled, or TAT alone -peptide transduced cells. The data shown are the mean ± SD of three different immunoblots. (D) The effect of sNT-LPL peptides on F-actin content: The F-actin content was determined in osteoclasts transduced with indicated peptides by rhodamine-phalloidin binding. Fold change in F-actin levels are provided. The F-actin content of the 0-min cells was assigned a value of 1.0, and all other values were expressed relative to the 0-mins values. Values plotted are mean±SD from three experiments **p<0.01; *p<0.05 versus P2, P5, or P6 peptide transduced cells.

In this context, we tested whether an sNT-LPL peptide containing Ser-5 and Ser-7 (1MARGSVSDEE10) can be used as an inhibitor of endogenous (cellular) LPL phosphorylation and NSZs formation. Therefore, we generated the following sNT-LPL peptides (denoted as P1-P6) as shown in Fig 1A: Unsubstituted (P1), Ser- 5 and Ser-7 substituted to Alanine 5, and 7 (P2; A5A7), either Ser-5 (P3; A5S7) or Ser- 7 (P4; S5A7) is substituted to Alanine and scrambled (P5). TAT peptide (P6) alone was also used as a control besides P2 (Ser5-Ser-7 substituted to Ala) and P5 (scrambled) peptides.

First, we determined the time-dependent uptake of sNT-LPL peptides indicated in Fig 1A using immunostaining analysis (S1 Fig). Immunostaining analysis was done with a TAT antibody and followed by confocal microscopy analyses. Fluorescent micrographs of transduced cells are illustrated at time points of 1, 6, 12, and 24h (S1 Fig). Diffuse staining was observed at all time points tested (Parts A-E in S1 Fig). The level was maintained until 10-14h and reduced after 20h. Staining with a species-specific non- or pre-immune serum was used to determine the background signal (Part F in S1 Fig). Based on the background staining with the NI serum, we suggest that immunostaining analysis with a TAT-antibody is specific.

Subsequently, immunoblotting of LPL immunoprecipitates was done with a p-Serine antibody (Fig 1B). Osteoclasts treated with bone particles and TNF-α were transduced with indicated peptides (Fig 1A) for 3-4h. Statistical analysis of the inhibition (%) of phosphorylation is shown in Fig 1C. No significant inhibitory effect was observed on the phosphorylation of cellular LPL in osteoclasts transduced with P2 (Fig 1B; lane 3), P5 (lane 6) and P6 (lane 7). However, different inhibitory effects are shown with peptides P1, P3, and P4 (lanes 2, 4, and 5). The inhibition is significant with P1 peptide (Fig 1B Lane 2; Fig 1C). Osteoclasts untransduced but treated with TNF-α and bone particles demonstrated basal level phosphorylation (lane 8) equal to the P2, P5, or P6 peptide transduced cells. Lysate made from osteoclasts transduced with P2 was used for immunoprecipitation with non-immune serum (lane 1). The levels of LPL protein in each immunoprecipitate are shown after stripping and reblotting with an antibody to LPL (Fig 1B; LPL). We showed here that P1, P3, and P4 peptides have the potential to competitively suppress the serine phosphorylation of endogenous LPL in the following order P1>P3 = P4. Immunoblotting of total lysates with an antibody to GAPDH validates that an equal amount of protein was used for immunoprecipitation (Fig 1B).

Changes in F-actin levels is considered as a marker for cellular structural transformation. The transformation of the actin cytoskeleton from podosome organization to sealing rings comprises both fundamental and quantitative modifications in resorbing osteoclasts. Rhodamine-phalloidin is a highly specific probe for filamentous actin. Therefore, to determine the quantitative changes in the F-actin levels in osteoclast treated with indicated peptides, we used the rhodamine–phalloidin binding assay as described [22]. F-actin level decreased in osteoclasts treated with P1, P3, and P4 (Fig 1D) as compared with P2, P5, and P6. The decrease was significant with P1 peptide (Fig 1D) similar to the results observed in the serine phosphorylation of cellular LPL (Fig 1B and 1C). The % inhibition on the F-actin level was observed in the following order P1>P3 = P4>P2 = P5 = P6.

### Analyses of the effect of LPL peptides on osteoclast actin modulation (NSZs formation) and resorption of dentine matrix

**Fig 2:**
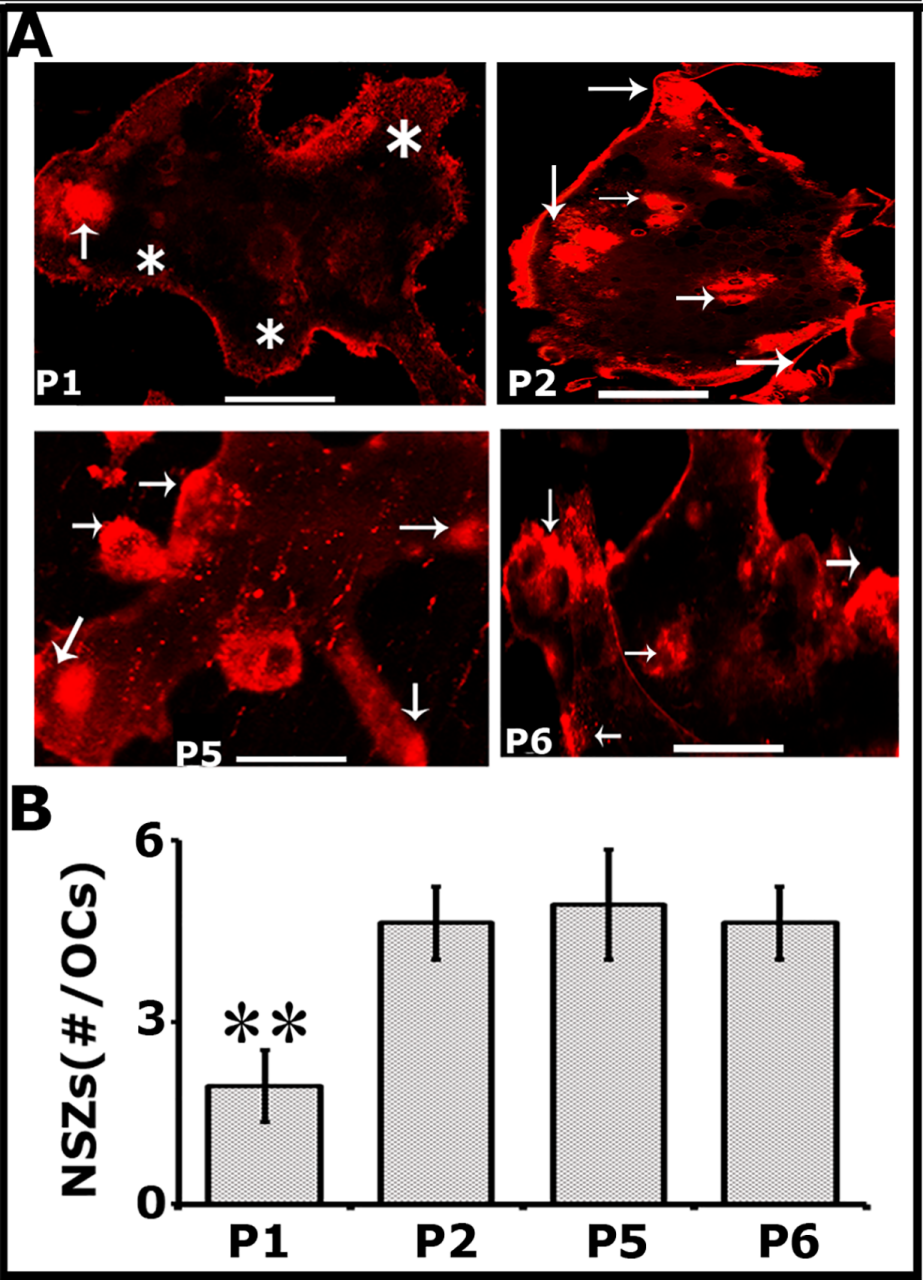
Peptide P1 (dominant negative) reduces the formation of TNF-α mediated NSZs formation considerably as compared with P2, P5, and P6 control peptides. Confocal microscopy analysis was done in osteoclasts stained for actin with rhodamine phalloidin (red). Arrows point to NSZs and Asterisks indicate podosome-like structures. Scale bar-25μm. The number of NSZs were counted in >100 osteoclasts and presented as #/OC in the graph (B). Data provided are mean±SD from one experiment. **p<0.01 versus mutated (P2), scrambled (P5), or TAT peptide (P6) transduced cells. Resorption assay was repeated several times with different osteoclast preparations and obtained similar results.

**Fig 3:**
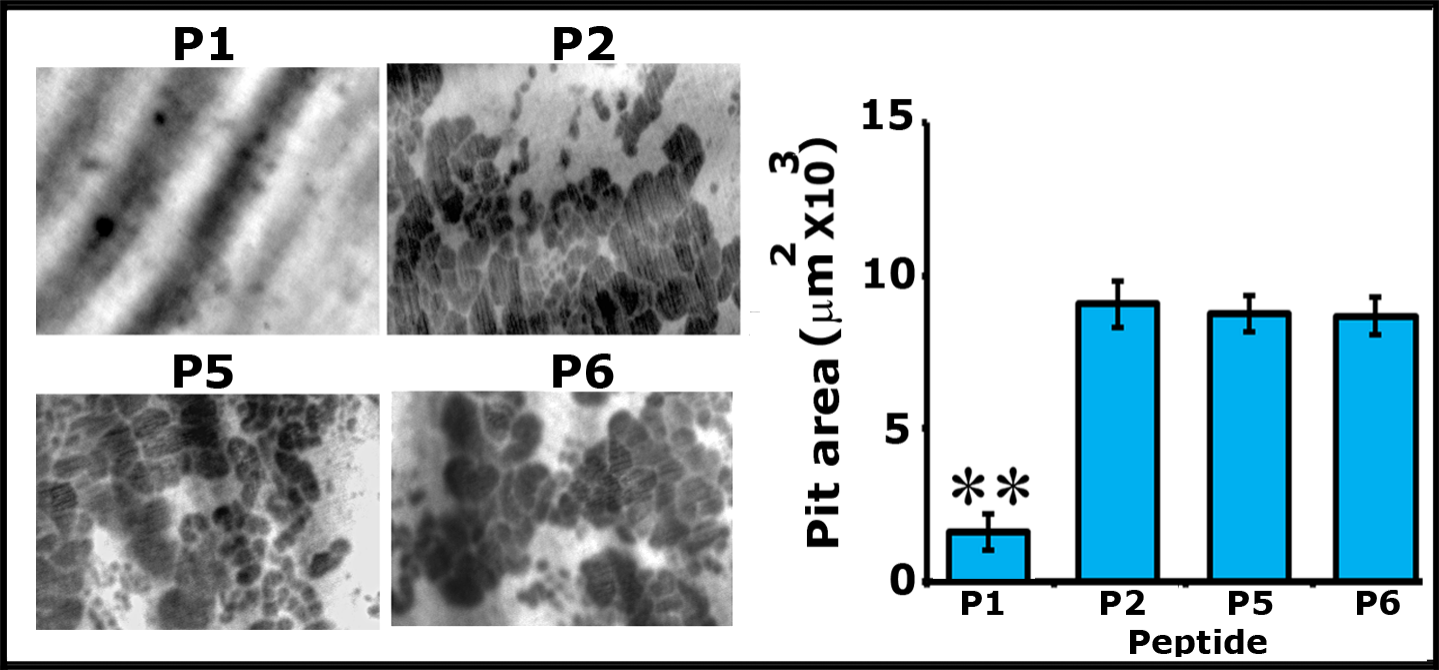
Peptide P1 reduces the resorption of dentine matrix considerably as compared with P2, P5, and P6 peptides. All treatments were done in triplicates, or quadruplicates for 12-16h and pits were viewed under 40X objective in a phase contrast microscope and photographed. Magnification is X400. The resorbed pit areas (20–25 pits/ slice) were quantified and data were compiled from four slices per treatment. The data shown are the mean ± SEM of one experiment performed (B). Experiments were repeated three times with three different osteoclast preparations. **p<0.001 versus mutated (P2), scrambled (P5), or TAT peptide (P6) transduced cells.

Peptide P1 decreased the phosphorylation of cellular LPL and F-actin considerably (Fig 1). Hence, to further define and highlight the impact of serine phosphorylation on the actin bundling process and osteoclast activity, we compared the effects of P1 with other peptides such as P2, P5, and P6 in the formation of NSZs (Fig 2) and dentine resorption (Fig 3). Peptides P2 and P5 act like the control TATpeptide (P6) with no inhibitory effect on the formation of NSZs (Fig 2) and resorption of dentine matrix (Fig 3). A considerable decrease in the organization and the number of NSZs was observed with the P1 peptide (Fig 2). Peptide P1 exhibited a dominant negative effect on TNF-α signaling mediated NSZs formation as compared with other control peptides. Resorption pits formed by osteoclasts treated with P1 peptide seem superficial and also are very less in number. The decrease in the number of NSZs reflected on the resorption activity of these osteoclasts (Fig 3) which infers the impact of the peptide on the formation of mature sealing rings.

### Analyses of the distribution of actin/L-plastin and integrin αv/L-plastin in osteoclasts transduced with P1 and P5 peptides

**Fig 4:**
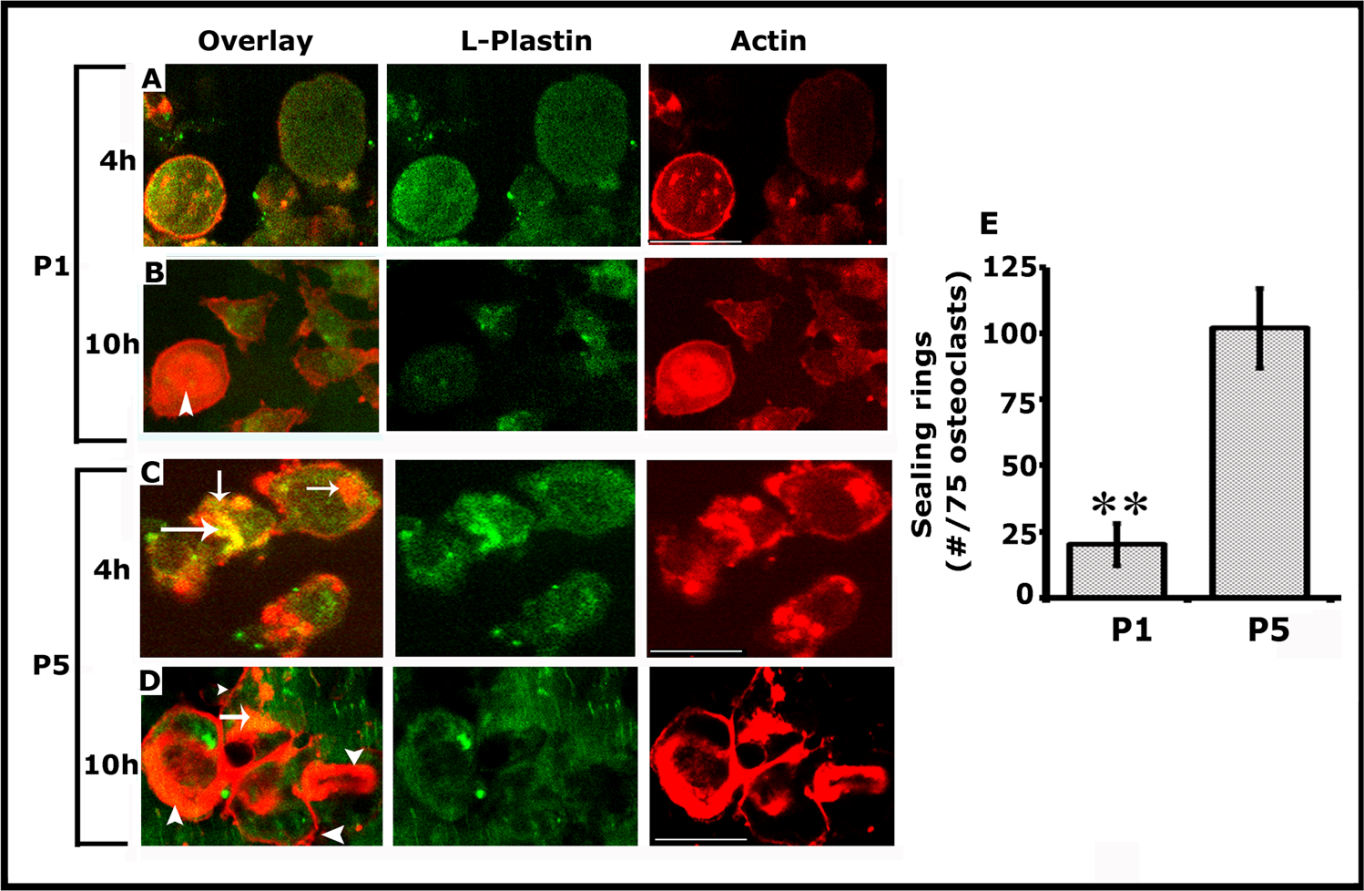
**Peptide P1 diminishes not only the formation of NSZs but also the sealing ring in osteoclasts cultured on dentine matrix for 4h (A and C) and 10h (B and D). A-D:** Immunostaining was done with an antibody to LPL (green); actin was stained with rhodamine phalloidin (red). Colocalization of actin and LPL (yellow; indicated by arrows) was observed in NSZs (C and D) in osteoclasts transduced with the P5 peptide. The mature sealing ring was observed from 10h onwards, and only actin (red) is present in the sealing ring (D; indicated by arrowheads). NSZ (A) and sealing ring (B) formation are significantly reduced in osteoclasts transduced with the P1 peptide. Diffuse distribution of LPL was observed in P1 and P5 tranduced osteoclasts. Scale bar: 150μm. **E.** Quantitative analysis of the number of sealing rings was done in approximately 75 osteoclasts and provided as a graph (Fig 4E). **p<0.001 versus scrambled (P5) transduced cells.

After transduction for 15-20min., cells were replated on dentine slices for 4h and 10h at which time we showed the formation of NSZs previously and sealing rings, respectively [3]. Here, we analyzed the distribution of actin/LPL in osteoclasts replated on dentine for 4 and 10h (Fig 4) and integrin αv/LPL in osteoclasts replated on dentine for 10h (S2 Fig). Confocal microscopy analyses corroborate our previous observations of the formation of NSZs at 4h and sealing rings at 10h (Fig 4A and 4B). Osteoclasts transduced with P5 peptide showed the formation of NSZs at 4h and sealing rings at 10h. A few of these NSZs indeed displayed colocalization of L-plastin and actin (yellow color; indicated by arrows in C and D); however, colocalization of LPL and actin is minimal or not observed in mature sealing rings although diffused LPL staining was observed at 10h (Fig 4D; green panel). Osteoclasts treated with P1 peptide demonstrated a significant decrease in the formation of NSZs at 4h (A) and mature sealing rings at 10h (B). Consistent with the observations shown in Fig 2, P1 peptide significantly reduced the formation of NSZs although a diffuse distribution of LPL was observed (Fig 4A). Attenuation of the formation of NSZs is reflected in the development of mature sealing rings (Fig 4B). An osteoclast which demonstrates a sealing ring is indicated by an arrow head in Fig B (Overlay). A quantitative analysis of the number of sealing rings was done in approximately 75 osteoclasts and provided as a graph (Fig 4E). A significant reduction in the formation of mature sealing rings by P1 peptide (Fig 4B) supports the bone resorption data provided in Fig 3.

The ultimate goal of these studies is to identify the effects of the P1 peptide on integrin-mediated sealing ring formation. Therefore, we proceeded to clarify further whether attenuation of NSZs formation by P1 also affects the localization and function of αvβ3 as well as the maturation of NSZs to sealing rings. Osteoclasts transduced with P1 and P5 (scrambled) peptides for 10h were immunostained with an LPL (green) and Integrin αv (red) antibody (S2 Fig). P1 peptide attenuates the NSZs (Fig 4) and therefore sealing ring formation (S2 Fig; P1). Diffused distribution of LPL (green) and integrin αv (red) was observed in these osteoclasts. But, these proteins are not colocalized. Colocalization of LPL/ Integrin αv was noted in a few of the developing sealing rings (indicated by arrowheads in P1-overlay panel). However, osteoclast transduced with a P5 peptide demonstrated several mature sealing rings (indicated by arrows) P5; red panel). Colocalization of LPL/ Integrin αv was not observed in sealing rings. These observations were in line with our previous study showing that NSZs function as a hub for orchestrating integrin signaling [3]. LPL phosphorylation is critical in the actin bundling process required for the formation of NSZs. The fact that suppression of LPL phosphorylation by the P1 peptide is entirely consistent with the role LPL at the early stage of sealing ring formation. Reduced resorption of dentine in osteoclasts transduced with P1 peptide (Fig 3) is apparently due to the attenuation of sealing ring formation.

### Analyses of the effects of peptides on the migration and podosome assembly

LPL was shown to present in the podosomes of osteoclasts [11]. Podosomes are implicated in the migration of osteoclasts [2, 17, 26]. Having observed that P1 peptide has significant inhibitory effects on the formation of NSZs, we examined whether it would have a similar effect on podosome assembly and the migration of osteoclasts (S3 Fig). Migration was assessed using phagokinesis and Corning transwell migration assays (Parts A-C in S3 Fig). Neither the migration (A-C) nor the formation of the podosomes (D) was affected by LPL peptides (P1, P2, P5, and P6). Our results point to the importance of LPL phosphorylation in the actin bundling process required for the formation of NSZs. This process is reduced in osteoclasts transduced with the P1 peptide. Further studies are needed to identify the signaling mechanisms involved in LPL phosphorylation. How the phosphorylation of LPL is regulated in response to TNF-α and bone particles is currently under study.

### Analyses in osteoclasts derived from RAW cells

**Fig 5:**
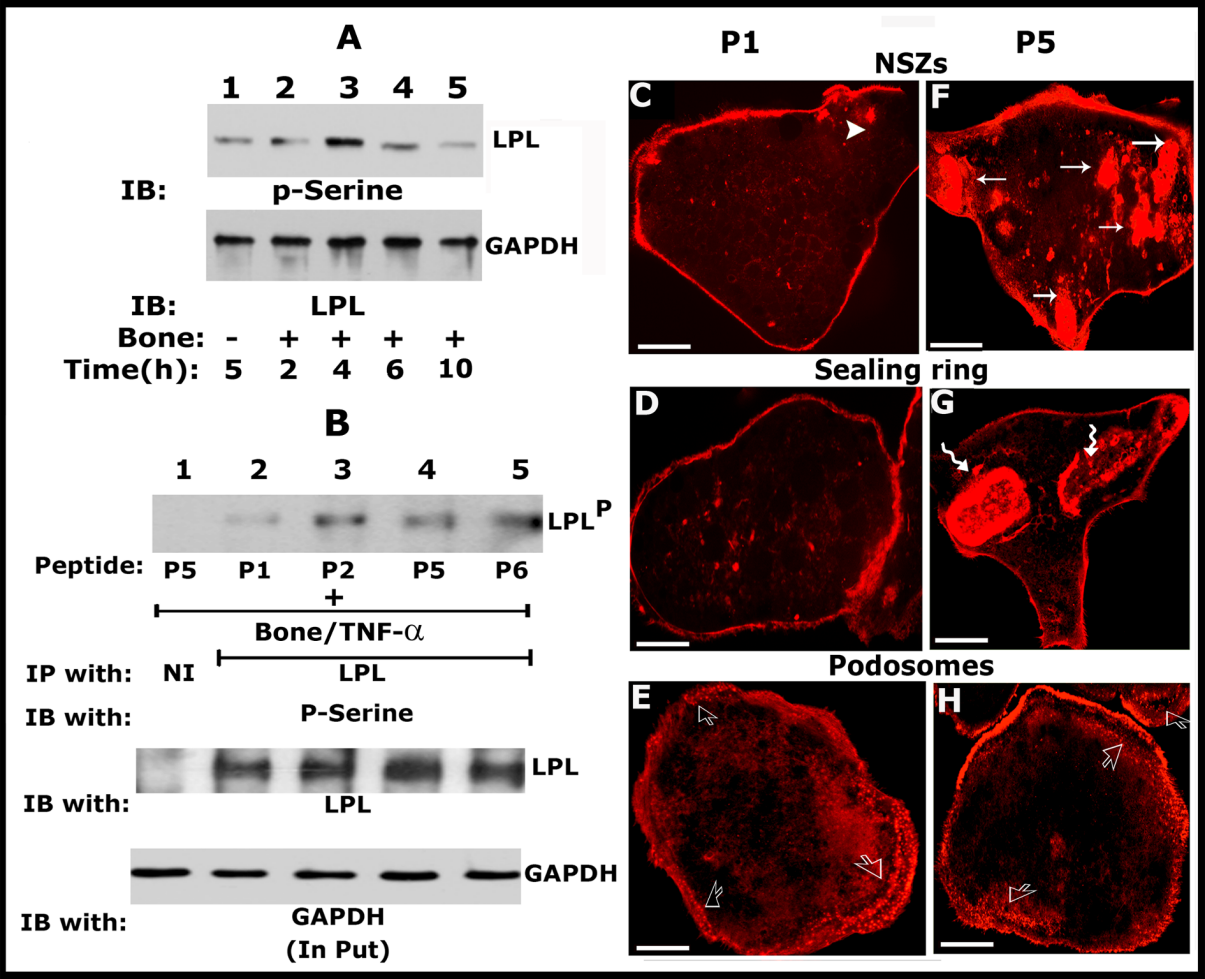
Analyses in osteoclasts derived from RAW macrophage cell line corroborates the observations in mouse BM cells. (A) Immunoblotting (IB) with an LPL antibody: Osteoclasts derived from RAW cells were incubated with bone particles (+) and TNF-α for different time periods (h) as indicated at the bottom of the figure. Immunoblotting analyses with an LPL antibody is shown (Panel A; top). This blot was stripped and blotted with a GAPDH antibody (bottom). Osteoclasts untreated with bone particles but treated with TNF-α were used as controls (-; lane 1). Osteoclasts treated with bone particles (+) and TNF-α for an indicated time–period (h) are shown in lanes 2–5. (B) Immunoprecipitation and immunoblotting analyses: Equal amount of osteoclast lysates were immunoprecipitated with an antibody to LPL and subjected to IB with a p-Serine antibody (Panel B; top). This blot was stripped and blotted with an LPL antibody (Panel B; middle). An equal amount of protein used for immunoprecipitation was assessed by direct immunoblotting of total lysates (input) with a GAPDH antibody (bottom). These results represent one of the three experiments performed with the similar results. (C) Confocal microscopy analyses in osteoclasts treated with peptides (P1 and P5) and stained for actin with rhodamine phalloidin (red): Arrows point to NSZs (F) and wavy arrows point to sealing rings (G) in P5 peptide treated osteoclasts plated on dentine slices. These features are significantly reduced or not observed in P1 peptide treated osteoclasts (C and D). An arrowhead in C points to a small actin aggregate. Open arrows in E and H point to podosomes in P1 and P5 treated osteoclasts plated on glass coverslips. Scale bar-25μm.

We validated the effects of LPL peptides on LPL phosphorylation and actin modulation in osteoclasts derived from the RAW macrophage cell line. Consistent with the observations shown in mouse BM-derived osteoclasts [3], LPL level is maximum at 4h (Fig 5A; top panel, lane 3) and decreased from 6h onwards (Lanes 4 and 5) in RAW cells derived osteoclasts treated with bone particles and TNF-α. Immunoblotting of the same blot with the GAPDH was used as a loading control (Fig 5A; bottom panel). We then tested the effect of P1, P2, P5, and P6 peptides on the phosphorylation of endogenous LPL by immunoprecipitation and immunoblotting analyses (Fig 5B). We used P1 and P5 (scrambled) peptides to evaluate the organization of actin filaments in resorbing (C, D, F, and G) and non-resorbing (E and H) osteoclasts. Osteoclasts were stained with phalloidin to visualize filamentous actin in confocal microscopy. The finding of a significant decrease in the phosphorylation of cellular LPL (Fig 5B, lane 2) and the formation of NSZs (Fig 5C-P1) and sealing rings (Fig 5D-P1) by P1 in osteoclasts derived from RAW cells well corroborates with the P1 peptide effects in mouse BM-derived osteoclasts (Figs 1 and 2). The P1 peptide which is having a significant impact on the formation of NSZs does not affect the pattern of podosome organization (Fig 5E) as compared with P5 peptide (Fig 5H).

### Analyses of the effect of transduction of TAT-fused LPL peptides on osteoblast function

**Fig 6:**
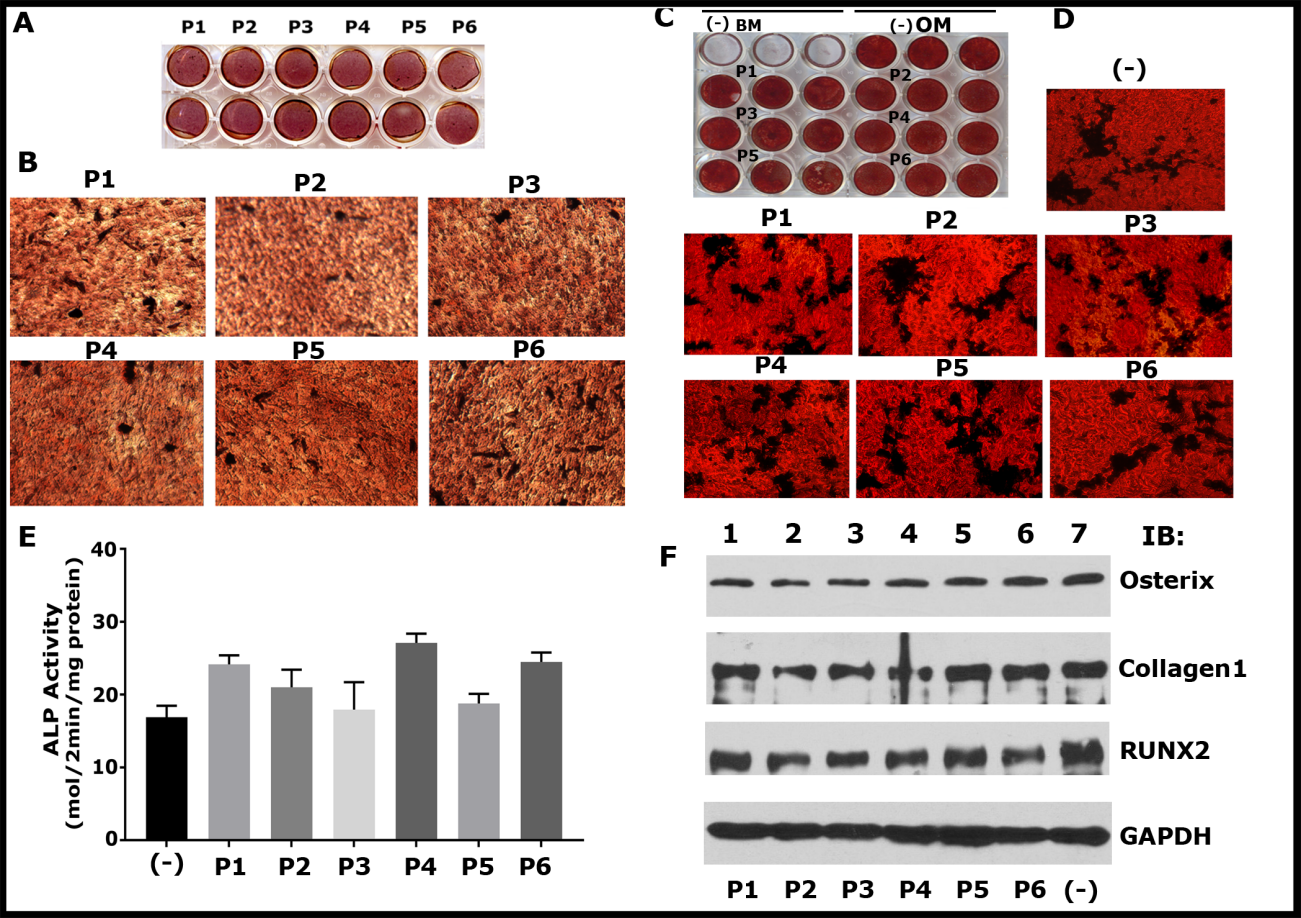
Mineralization and expression of osteogenic markers (Collagen, RUNX2, and Osterix) by osteoblasts are unaffected by sNT-LPL peptides (P1-P6). (A-D) Mineralization was assessed by Alizarin Red Staining (ARS) in cells fixed at day 7 of culture. ARS of MC3T-E1 (A and B) and UMR-106 (C and D) cells cultured for seven days is shown. Each peptide treatment was done in duplicates for MC3T3 (A) and triplicates for UMR-106 (C) cells. The plates were scanned in an EPSON Perfection V200 Photo scanner (A and C). Representative magnified images of mineralized nodules are shown (B and D). Magnified images were taken in a phase contrast microscopy with a 10X and 20X objective for B and D, respectively. Cells were grown in osteogenic medium (OM), and basal medium (BM) without peptides (-) were used as controls. (E) Analysis of ALP activity in UMR 106 cells. Data shown are mean ± SD (n = 3). Minus (-) in C indicates cells grew in OM but untreated with the peptide. There is no significant statistical difference between the groups. (F) Western blot analysis for the expression of osteogenic biomarkers such as Collagen 1, RUNX2 and osterix. Lysates made from osteoblasts treated with indicated peptides for seven days were used for the analysis. Immunoblotting with a GAPDH antibody was used as loading control. Results in A-D and F represent one of the two experiments performed with the similar results.

Osteoblasts are the cells that make bone, and these cells do not express LPL [27]. The study has been extended to assess the effects of these peptides (P1-P6) on the mineralization process arbitrated by osteoblasts derived from MC3T3 (Fig 6A and 6B) and UMR-106 cells (Fig 6C–6E). We used the following analyses: ARS staining and ALP activity assay to determine the formation of mineralized matrix (Fig 6A–6E) and immunoblotting studies to assess the expression of osteogenic biomarkers (Collagen 1, Osterix, and RUNX2; Fig 6F). P1-P6 peptides did not affect the formation of the mineralized matrix (Fig 6A–6E) and the expression of osteogenic biomarkers for bone formation (Fig 6F).

**Fig 7:**
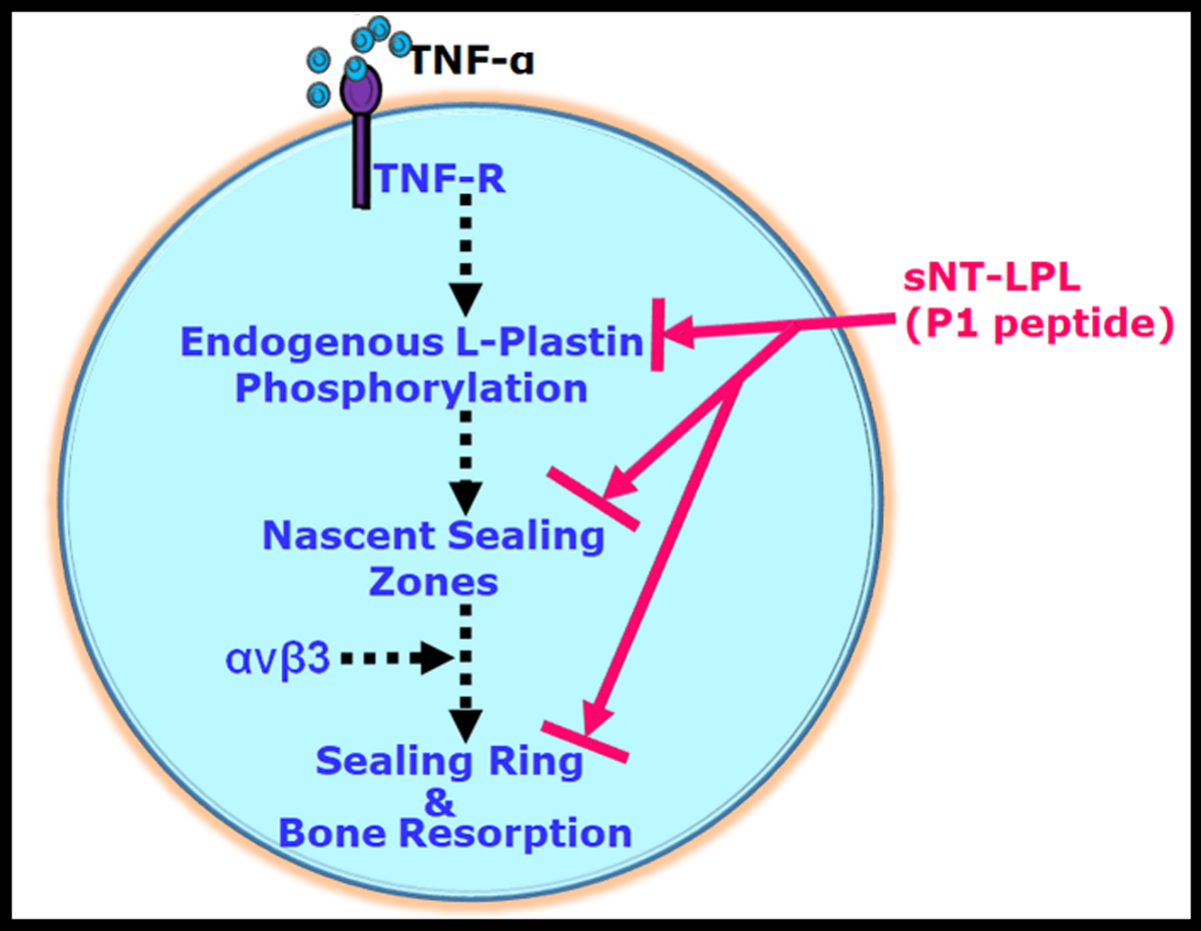
The proposed model of the role of L-Plastin (LPL) in nascent sealing zones (NSZs) formation. TNF-α signaling regulates the phosphorylation of LPL on serine residues which mediate the actin bundling process involved in the formation of NSZs. Osteoclasts treated with an inhibitor to αv or siRNA to β3 reduced the formation of matured sealing rings but not NSZs [ref. 3]; broken arrows). Therefore, NSZ formation occurs independently of integrin αvβ3 signaling. We show in this paper that sNT-LPL (P1) peptide has the potential to block the serine phosphorylation of endogenous LPL competitively. Inhibition of phosphorylation attenuates the actin bundling process involved in NSZs formation. Failure of formation of NSZs has an impact on the formation of sealing rings formation and bone resorption (solid arrows). We suggest that these NSZs are the presumable precursor zones for fully functional sealing rings. LPL is a possible attractive target for pharmacologic regulation of osteoclast sealing ring formation via blocking the formation of NSZs by LPL.

Overall, our data demonstrate the critical role of LPL in actin bundling process involved in the organization of NSZs. NSZs are the presumed precursor zones for sealing rings. TNF-α was shown to stimulate the resorptive activity of osteoclasts independently of αvβ3 [28,29]. The actual target of TNF-α signaling has not been identified. We have previously shown that a neutralizing antibody reduces LPL phosphorylation to TNF-α and TNF-receptor 1 (TNFR1). The formation of NSZs seems dependent on TNF-α signaling [3]. A decrease in NSZ formation in anti- TNFR1 treated cells suggests a role for TNF-α signaling in this process independently of αvβ3. Phosphorylation of LPL and the formation of NSZs by TNF-α signaling may be key elements at the early stage of sealing ring formation. We showed here the peptide’s (peptide P1) ability to enter into osteoclasts and suppress the phosphorylation of endogenous LPL competitively and hence the formation of NSZs and fully functional mature sealing rings. Failure in the formation of sealing rings inhibits the bone resorption by osteoclasts (Fig 7). These observations justify testing the effect of peptides in vivo in animal models of osteoporosis. The limitation here is that the molecular mechanisms involved in the assembly of NSZs have not been studied. Further experiments are required to determine the critical signaling molecules in the TNF-α signaling pathway which regulates LPL phosphorylation.

## Discussion

The mechanism by which osteoclasts coordinate sealing ring formation is not clear. We have previously shown that cyclical changes in protein and phosphorylation levels of LPL and cortactin correspond with time-dependent changes in actin organization in osteoclasts subjected to bone resorption. LPL was shown to localize in the podosomes of monocytes derived osteoclasts [11]; however, little is known about its function. We were the first to have shown the assembly of actin aggregates at the early stage of sealing ring formation by TNF-α or RANKL signaling independent of integrin αvβ3 signaling. These actin aggregates are denoted as nascent sealing zones (NSZs) [3]. Osteoclasts derived from RAW cells corroborate our previous observations that polymerization of actin generates a force to push the plasma membrane forward to produce membrane extensions. NSZs formed at the extensions serve as adhesive structures which facilitate the spreading of osteoclasts on bone. NSZs are considered the presumable precursors for sealing rings. Expression and phosphorylation of LPL assists in the process of NSZ formation. Studies in T-lymphocytes have shown that LPL expression and phosphorylation at Ser- 5 influences the localization of F-actin cytoskeletal protein and cellular polarization in response to chemokine stimulation [30]. T cells deficient in LPL are defective in cellular polarization [31]. This suggests that LPL is a key regulator of T-cell receptor-mediated actin rearrangement required for the cellular polarization.

Osteoclasts polarize during bone resorption. It is not completely known whether LPL has any role in the polarization of osteoclasts on dentine or bone during resorption. It seems LPL plays a different role in osteoclasts. LPL is a crucial regulator of the actin bundling process which is required for sealing ring formation. Osteoclasts plated on dentine slices and treated with TNF-α displayed an actin cytoskeleton composed of NSZs which are the organization zone for sealing ring and subsequent formation of one or a few mature sealing rings. During the early stages of the maturation of sealing ring, actin patches are converted to ring-like configurations [3].

L-plastin is phosphorylated on residues Ser-5 and Ser- 7 in hematopoietic cells in vivo, but most likely on Ser-5 on non-hematopoietic cells. Phosphorylation of Ser-5 residue upstream of cytoskeletal rearrangements that underlie processes such as chemotaxis and adhesion [10, 12, 13]. Serine phosphorylation may have a direct role in the actin bundling function of LPL when expressed ectopically in Vero cells [24]. LPL stabilizes actin filaments and protects them against depolymerization [16]. The physiological function of Ser-7 phosphorylation is not known [4, 15]. To elucidate the role of LPL phosphorylation in NSZ formation, we have used sNT-LPL peptides (10aa). Peptide P1 exerts inhibitory effects on the function of cellular LPL as assayed by the levels of phosphorylation of cellular LPL, changes in actin dynamics and the capacity to resorb bone. Peptide P1 competitively reduced the function of cellular LPL more than the P3 and P4 with amino acid substitution at either Ser-5 or Ser-7. These results indicate that the phosphorylation of both Ser-5 and Ser-7 residues is important in LPL- mediated effects in osteoclasts. Inhibition of phosphorylation of cellular LPL reduced the actin bundling process mediated by the ABDs of cellular LPL. Our results indicate that cooperativity between serine phosphorylation and actin binding to ABDs is required for the actin bundling process mediated by LPL.

As shown in neutrophils, regulation of actin bundling process by LPL is related to its phosphorylation on the serine residues [32]. Integrin αvβ3 plays a vital role in the adhesion of osteoclasts on bone matrix and regulation of cytoskeletal organization essential for the formation of sealing rings [33–35]. Since Integrin αvβ3 is associated with several signaling molecules involved in the formation (‘maturation’) of sealing rings, its localization in the NSZs is vital. We previously reported that localization of integrin in the NSZs is LPL- dependent and the maturation of the sealing rings from NSZs are under the regulation of integrin αvβ3 signaling [3]. Due to the architectural nature of sealing rings, the major reorganization of actin filaments is required during their formation. Sealing rings consisting of stable actin filaments generate tight sealing zones on the bone surface. NSZs formed by LPL function as a central point or a hub in assembling molecular components (integrin αvβ3, Src, cortactin, ERK, WASP, and Arp2/3) involved in the maturation of NSZs to fully functional mature sealing rings [3, 36, 37]. Time-dependent changes in the localization of LPL in NSZs and cortactin in sealing rings suggest that these proteins may be involved in the initial and maturation phases of sealing rings, respectively [37]. The failure of the P1 peptide to bring about NSZs and sealing ring organization also support our previous observations of the role LPL phosphorylation on the recruitment of integrin and associated signaling molecules in NSZs for the maturation process [3]. LPL peptide was shown to synergize with RGD ligand for the generation of conformational changes associated with the high-affinity state of integrin αvβ3 in PMNs [38]. However, we show here that the P1 peptide functions as a competitive inhibitor of cellular or intracellular LPL-mediated actin dynamics.

The role of LPL appears to be cell-type specific in the localization of integrin αvβ3 in PMNs [38] and podosome formation in macrophages. We suggest this because phosphorylated L-plastin is enriched in podosomes where it colocalizes with F-actin, consistent with the idea that its phosphorylation enhances F-actin binding and bundling [39]. LPL was shown to be a vital protein for podosome formation and function in macrophages. Enrichment of phosphorylated LPL in podosomes emphasizing the actin bundling property of LPL in podosome stability. Expression of nanobodies generated against distinct domains of LPL perturbed matrix degradation, migration, podosome formation/ steadiness in THP-1 macrophages [40]. LPL was shown to present in the podosomes of osteoclasts [11]; however little is known about the functional consequences of its expression. The P1 peptide which reduces the formation NSZs and sealing rings does not affect podosome formation or migration of osteoclasts. Superficial pits observed in P1 treated osteoclasts may be due to the degradation of matrix proteins by MMPs present in the podosomes during migration [41, 42]. Podosomes of highly invasive and migratory cells are linked with adhesive/migratory functions accompanied by proteolytic degradation of the extracellular matrix [43–45].

Plastin 3 is expressed in osteoblasts. Mutations in plastin 3 resulted in osteoporosis in mice which signifies the role of plastin 3 in osteoblasts and not osteoclasts [46]. Inhibition of bone resorption and not formation by P1 peptide highlight the essentiality of LPL (aka plastin 2) in osteoclast sealing ring formation. The findings herein demonstrate the significance of LPL phosphorylation and function in NSZ formation at the early stage of sealing ring formation. Attenuation of NSZ formation in osteoclasts transduced with P1 peptide corroborates the importance of phosphorylation of LPL in osteoclast cytoskeletal remodeling involved in bone resorption.

## Conclusions

Based on the results with LPL peptide, we believe that LPL not only has the potential to organize the actin bundling process involved in the formation of NSZs but also the stability of bundled F-actin for the maturation processes involved in the formation of sealing rings. Maturation is mediated by integrin αvβ3 signaling which comprises several signaling, actin-binding, and regulatory proteins [3, 20, 47–50]. Within this study, we identify LPL as a novel therapeutic target in osteoclast-mediated events. Also, sNT-LPL (P1) based manipulations of osteoclast bone resorption have the potential for pharmacological manipulations. We conclude that LPL is indispensable to drive the actin bundling processes involved in sealing ring formation.

## Supporting information

S1 Fig Time-dependent uptake of indicated TAT-fused sNT-LPL peptides (P1-P5) by osteoclasts. Confocal microscopy analysis of osteoclasts immunostained with a TAT- antibody (A-E) and a non-immune serum (F) is shown. TAT-stained osteoclasts at different times (1, 6, 12, and 24h) after transduction with indicated TAT-fused peptides are shown. Scale bar-150 μm.
(TIF)

S2 Fig Confocal microscopy analyses of osteoclasts immunostained for integrin αv (red) and L-plastin (green). Osteoclasts transduced with P1 and P5 (scrambled) peptides for 10h were immunostained with an LPL (green) and Integrin αv (red) antibody. Sealing rings are indicated by arrows (P5; red panel). Arrowheads point to NSZs developing into mature sealing rings which demonstrate colocalization (yellow) of LPL (green) and integrin αv (red) (P1; overlay panel). Scale bar: 150μm.
(TIF)

S3 Fig Migration and podosome organization are unaffected by sNT-LPL peptides in mouse osteoclasts. Motility (A-C) and podosome organization (D) was assessed in osteoclasts treated with peptides (P1, P2, P5, and P6). (A-C) Phagokinesis (A and B) and transwell migration (C) assays. The data in B are mean ± SD of 20–30 cell tracks (clear areas) represented as area migrated in mm^2^. The data in C are mean ± SD of migrated cells and provided as cells/filed. Images were taken in a phase contrast microscopy with a 10X objective (magnification X100). (D) Confocal microscopy analysis of rhodamine-phalloidin stained cells. None of the indicated peptides has any effect on osteoclast migration (A-C) or podosome organization (D). Podosome organization is seen at the periphery of osteoclasts. These results represent one of the three experiments performed with the similar results. Scale bar: 50μm.
(TIF)

## Abbreviations

ABP: actin-binding protein
RANKL: receptor activator of NF-κB ligand
M-CSF: macrophage colony stimulating factor
LPL: L-Plastin
sNT-LPL: small molecular weight amino terminal L-plastin peptide
NT-LPL: amino-terminal L-plastin peptide
TNF-α: tumor necrosis factor alpha
TAT: transactivator peptide with transforming properties
ABDs: actin binding domains
PMN: polymorphonuclear neutrophils

## References

[1] Zambonin-Zallone A, Teti A, Carano A, Marchisio PC. The distribution of podosomes in osteoclasts cultured on bone laminae: effect of retinol. J Bone Miner Res 1988;3:517–23. 10.1002/jbmr.5650030507 3195364

[2] Chellaiah M, Soga N, Swanson S, McAllister S, Alvarez U, Wang D, et al Rho-A is critical for osteoclast podosome organization, motility, and bone resorption. Journal of Biological Chemistry 2000;275:11993–2002. 10766830 10.1074/jbc.275.16.11993

[3] Ma T, Sadashivaiah K, Chellaiah MA. Regulation of sealing ring formation by L-plastin and cortactin in osteoclasts. J Biol Chem 2010 9 24;285(39):29911–24. 10.1074/jbc.M109.099697 20650888 PMC2943304

[4] Lin CS, Lau A, Lue TF. Analysis and mapping of plastin phosphorylation. DNA Cell Biol 1998 12;17(12):1041–6. 10.1089/dna.1998.17.1041 9881671

[5] Delanote V, Vandekerckhove J, Gettemans J. Plastins: versatile modulators of actin organization in (patho)physiological cellular processes. Acta Pharmacol Sin 2005 7;26(7):769–79. 10.1111/j.1745-7254.2005.00145.x 15960882

[6] Namba Y, Ito M, Zu Y, Shigesada K, Maruyama K. Human T cell L-plastin bundles actin filaments in a calcium-dependent manner. J Biochem (Tokyo) 1992 10;112(4):503–7. 1491005 10.1093/oxfordjournals.jbchem.a123929

[7] Bretscher A. Purification of the intestinal microvillus cytoskeletal proteins villin, fimbrin, and ezrin. Methods Enzymol 1986;134:24–37. 3821564 10.1016/0076-6879(86)34072-2

[8] Arpin M, Friederich E, Algrain M, Vernel F, Louvard D. Functional differences between L- and T-plastin isoforms. J Cell Biol 1994 12;127(6 Pt 2):1995–2008. 7806577 10.1083/jcb.127.6.1995 PMC2120298

[9] Jones SL, Wang J, Turck CW, Brown EJ. A role for the actin-bundling protein L-plastin in the regulation of leukocyte integrin function. Proc Natl Acad Sci U S A 1998 8 4;95(16):9331–6. 9689080 10.1073/pnas.95.16.9331 PMC21338

[10] Chen H, Mocsai A, Zhang H, Ding RX, Morisaki JH, White M, et al Role for plastin in host defense distinguishes integrin signaling from cell adhesion and spreading. Immunity 2003 7;19(1):95–104. 12871642 10.1016/s1074-7613(03)00172-9

[11] Babb SG, Matsudaira P, Sato M, Correia I, Lim S-S. Fimbrin in podosomes of monocyte-derived osteoclasts. Cell Motil Cytosk 1997;37:308–25. 10.1002/(SICI)1097-0169(1997)37:4<308::AID-CM3>3.0.CO;2-0 9258504

[12] Jones SL, Brown EJ. FcgammaRII-mediated adhesion and phagocytosis induce L-plastin phosphorylation in human neutrophils. J Biol Chem 1996 6 14;271(24):14623–30. 8663066 10.1074/jbc.271.24.14623

[13] Foran E, McWilliam P, Kelleher D, Croke DT, Long A. The leukocyte protein L-plastin induces proliferation, invasion and loss of E-cadherin expression in colon cancer cells. Int J Cancer 2006 4 15;118(8):2098–104. 10.1002/ijc.21593 16287074

[14] Shibata M, Ohoka T, Mizuno S, Suzuki K. Characterization of a 64-kd protein phosphorylated during chemotactic activation with IL-8 and fMLP of human polymorphonuclear leukocytes. I. Phosphorylation of a 64-kd protein and other proteins. J Leukoc Biol 1993 7;54(1):1–9. 8393062 10.1002/jlb.54.1.1

[15] Otsuka M, Kato M, Yoshikawa T, Chen H, Brown EJ, Masuho Y, et al Differential expression of the L-plastin gene in human colorectal cancer progression and metastasis. Biochem Biophys Res Commun 2001 12 14;289(4):876–81. 10.1006/bbrc.2001.6047 11735128

[16] Lebart MC, Hubert F, Boiteau C, Venteo S, Roustan C, Benyamin Y. Biochemical characterization of the L-plastin-actin interaction shows a resemblance with that of alpha-actinin and allows a distinction to be made between the two actin-binding domains of the molecule. Biochemistry 2004 3 9;43(9):2428–37. 10.1021/bi030151p 14992580

[17] Chellaiah M, Kizer N, Silva M, Alvarez U, Kwiatkowski D, Hruska KA. Gelsolin deficiency blocks podosome assembly and produces increased bone mass and strength. J Cell Biol 2000;148:665–78. 10684249 10.1083/jcb.148.4.665 PMC2169374

[18] Chellaiah MA, Kuppuswamy D, Lasky L, Linder S. Phosphorylation of a Wiscott-Aldrich syndrome protein-associated signal complex is critical in osteoclast bone resorption. J Biol Chem 2007 3 30;282(13):10104–16. 10.1074/jbc.M608957200 17283076

[19] Gupta A, Lee BS, Khadeer MA, Tang Z, Chellaiah M, Abu-Amer Y, et al Leupaxin is a critical adaptor protein in the adhesion zone of the osteoclast. J Bone Miner Res 2003 4;18(4):669–85. 10.1359/jbmr.2003.18.4.669 12674328

[20] Chellaiah MA, Biswas RS, Rittling SR, Denhardt DT, Hruska KA. Rho-dependent Rho kinase activation increases CD44 surface expression and bone resorption in osteoclasts. J Biol Chem 2003 8 1;278(31):29086–97. 10.1074/jbc.M211074200 12730217

[21] Chellaiah M, Fitzgerald C, Alvarez U, Hruska K. C-*src* is required for stimulation of gelsolin associated PI3-K. Journal of Biological Chemistry 1998;273:11908–16. 9565618 10.1074/jbc.273.19.11908

[22] Chellaiah M, Hruska KA. Osteopontin stimulates gelsolin associated phosphoinositide levels and PtdIns 3-hydroxyl kinase. Mol Biol Cell 1996;7:743–53. 8744948 10.1091/mbc.7.5.743 PMC275927

[23] Delanote V, Vandekerckhove J, Gettemans J. Plastins: versatile modulators of actin organization in (patho)physiological cellular processes. Acta Pharmacol Sin 2005 7;26(7):769–79. 10.1111/j.1745-7254.2005.00145.x 15960882

[24] Al TZ, Schaffner-Reckinger E, Halavatyi A, Hoffmann C, Moes M, Hadzic E, et al Quantitative kinetic study of the actin-bundling protein L-plastin and of its impact on actin turn-over. PLoS ONE 2010;5(2):e9210 10.1371/journal.pone.0009210 20169155 PMC2821400

[25] Morley SC. The actin-bundling protein L-plastin supports T-cell motility and activation. Immunol Rev 2013 11;256(1):48–62. 10.1111/imr.12102 24117812 PMC3801223

[26] Chellaiah MA. Regulation of podosomes by integrin alphavbeta3 and Rho GTPase-facilitated phosphoinositide signaling. Eur J Cell Biol 2006 2 3;85((3–4)):311–7. 10.1016/j.ejcb.2006.01.008 16460838

[27] Li N, Wong CK, Cheng CY. Plastins regulate ectoplasmic specialization via its actin bundling activity on microfilaments in the rat testis. Asian J Androl 2016 9;18(5):716–22. 10.4103/1008-682X.166583 26608945 PMC5000794

[28] Fuller K, Murphy C, Kirstein B, Fox SW, Chambers TJ. TNFalpha potently activates osteoclasts, through a direct action independent of and strongly synergistic with RANKL. Endocrinology 2002 3;143(3):1108–18. 10.1210/endo.143.3.8701 11861538

[29] Fuller K, Kirstein B, Chambers TJ. Murine osteoclast formation and function: differential regulation by humoral agents. Endocrinology 2006 4;147(4):1979–85. 10.1210/en.2005-1340 16384864

[30] Freeley M, O'Dowd F, Paul T, Kashanin D, Davies A, Kelleher D, et al L-plastin regulates polarization and migration in chemokine-stimulated human T lymphocytes. J Immunol 2012 6 15;188(12):6357–70. 10.4049/jimmunol.1103242 22581862

[31] Morley SC, Wang C, Lo WL, Lio CW, Zinselmeyer BH, Miller MJ, et al The actin-bundling protein L-plastin dissociates CCR7 proximal signaling from CCR7-induced motility. J Immunol 2010 4 1;184(7):3628–38. 10.4049/jimmunol.0903851 20194718 PMC2855624

[32] Wang J, Brown EJ. Immune complex-induced integrin activation and L-plastin phosphorylation require protein kinase A. J Biol Chem 1999 8 20;274(34):24349–56. 10446213 10.1074/jbc.274.34.24349

[33] Miyauchi A, Alvarez J, Greenfield RM, Teti A, Grano M, Colucci S, et al Recognition of osteopontin and related peptides by alpha_v_beta_3_ integrin stimulates immediate cell signals in osteoclasts. Journal of Biological Chemistry 1991;266:20369–74. 1939092

[34] Nakamura I, Pilkington MF, Lakkakorpi PT, Lipfert L, Sims SM, Dixon SJ, et al Role of alpha(v)beta(3) integrin in osteoclast migration and formation of the sealing zone. J Cell Sci 1999 11;112 (Pt 22):3985–93. 10547359 10.1242/jcs.112.22.3985

[35] Duong LT, Rodan GA. Integrin-mediated signaling in the regulation of osteoclast adhesion and activation. Front Biosci 1998 8 1;3:d757–d768. 9682033 10.2741/A319

[36] Chellaiah MA, Schaller MD. Activation of Src kinase by protein-tyrosine phosphatase-PEST in osteoclasts: comparative analysis of the effects of bisphosphonate and protein-tyrosine phosphatase inhibitor on Src activation in vitro. J Cell Physiol 2009 8;220(2):382–93. 10.1002/jcp.21777 19350555 PMC7192836

[37] Ma T, Chellaiah MA. Dramatic inhibition of osteoclast sealing ring formation and bone resoprtion by a WASP-peptide containing pTyr294 aminoacid. J.Mol. Signal 2008 2 20; 3:(4). 10.1186/1750-2187-3-4 PMC2266921 18289379

[38] Wang J, Chen H, Brown EJ. L-plastin peptide activation of alpha(v)beta(3)-mediated adhesion requires integrin conformational change and actin filament disassembly. J Biol Chem 2001 4 27;276(17):14474–81. 10.1074/jbc.M007324200 11278342

[39] Janji B, Giganti A, De C, V, Catillon M, Bruyneel E, Lentz D, et al Phosphorylation on Ser5 increases the F-actin-binding activity of L-plastin and promotes its targeting to sites of actin assembly in cells. J Cell Sci 2006 5 1;119(Pt 9):1947–60. 10.1242/jcs.02874 16636079

[40] De CS, Boucherie C, Vandekerckhove J, Gettemans J, Guillabert A. L-plastin nanobodies perturb matrix degradation, podosome formation, stability and lifetime in THP-1 macrophages. PLoS ONE 2013;8(11):e78108 10.1371/journal.pone.0078108 24236012 PMC3827245

[41] Chellaiah MA, Hruska KA. The Integrin avb3 and CD44 Regulate the Actions of Osteopontin on Osteoclast Motility. Calcif Tissue Int 2002 12 10;72(3):197–205. 10.1007/s00223-002-1025-6 12469249

[42] Samanna V, Ma T, Mak TW, Rogers M, Chellaiah MA. Actin polymerization modulates CD44 surface expression, MMP-9 activation, and osteoclast function. J Cell Physiol 2007 12;213(3):710–20. 10.1002/jcp.21137 17508356

[43] Blavier L, Delaisse JM. Matrix metalloproteinases are obligatory for the migration of preosteoclasts to the developing marrow cavity of primitive long bones. J Cell Sci 1995 12;108 (Pt 12):3649–59. 8719871 10.1242/jcs.108.12.3649

[44] Sato T, del Carmen OM, Hou P, Heegaard AM, Kumegawa M, Foged NT, et al Identification of the membrane-type matrix metalloproteinase MT1-MMP in osteoclasts. J Cell Sci 1997 3;110 (Pt 5):589–96. 9092941 10.1242/jcs.110.5.589

[45] Desai B, Ma T, Chellaiah MA. Invadopodia and matrix degradation: a new property of prostate cancer cells during migration and invasion. J Biol Chem 2008 3 11; 283:13856–13866. 10.1074/jbc.M709401200 18337256 PMC2376228

[46] Fahiminiya S, Majewski J, Al-Jallad H, Moffatt P, Mort J, Glorieux FH, et al Osteoporosis caused by mutations in PLS3: clinical and bone tissue characteristics. J Bone Miner Res 2014 8;29(8):1805–14. 10.1002/jbmr.2208 24616189

[47] Duong LT, Rodan GA. The role of integrins in osteoclast function. J Bone Miner Metab 1999;17(1):1–6. 10084394 10.1007/s007740050055

[48] Miyazaki T, Sanjay A, Neff L, Tanaka S, Horne WC, Baron R. Src kinase activity is essential for osteoclast function. J Biol Chem 2004 4 23;279(17):17660–6. 10.1074/jbc.M311032200 14739300

[49] Teitelbaum SL. The osteoclast and its unique cytoskeleton. Ann N Y Acad Sci 2011 12;1240:14–7. 10.1111/j.1749-6632.2011.06283.x 22172034

[50] Saltel F, Chabadel A, Bonnelye E, Jurdic P. Actin cytoskeletal organisation in osteoclasts: a model to decipher transmigration and matrix degradation. Eur J Cell Biol 2008 9;87(8–9):459–68. 10.1016/j.ejcb.2008.01.001 18294724

