# Supplementary figures and images for: Peptidomimetic inhibitors of L-plastin reduce the resorptive activity of osteoclast but not the bone forming activity of osteoblasts in vitro
Source: PLoS One. 2018 Sep 24;13(9):e0204209. doi: 10.1371/journal.pone.0204209 (PMC6152981; doi:10.1371/journal.pone.0204209)

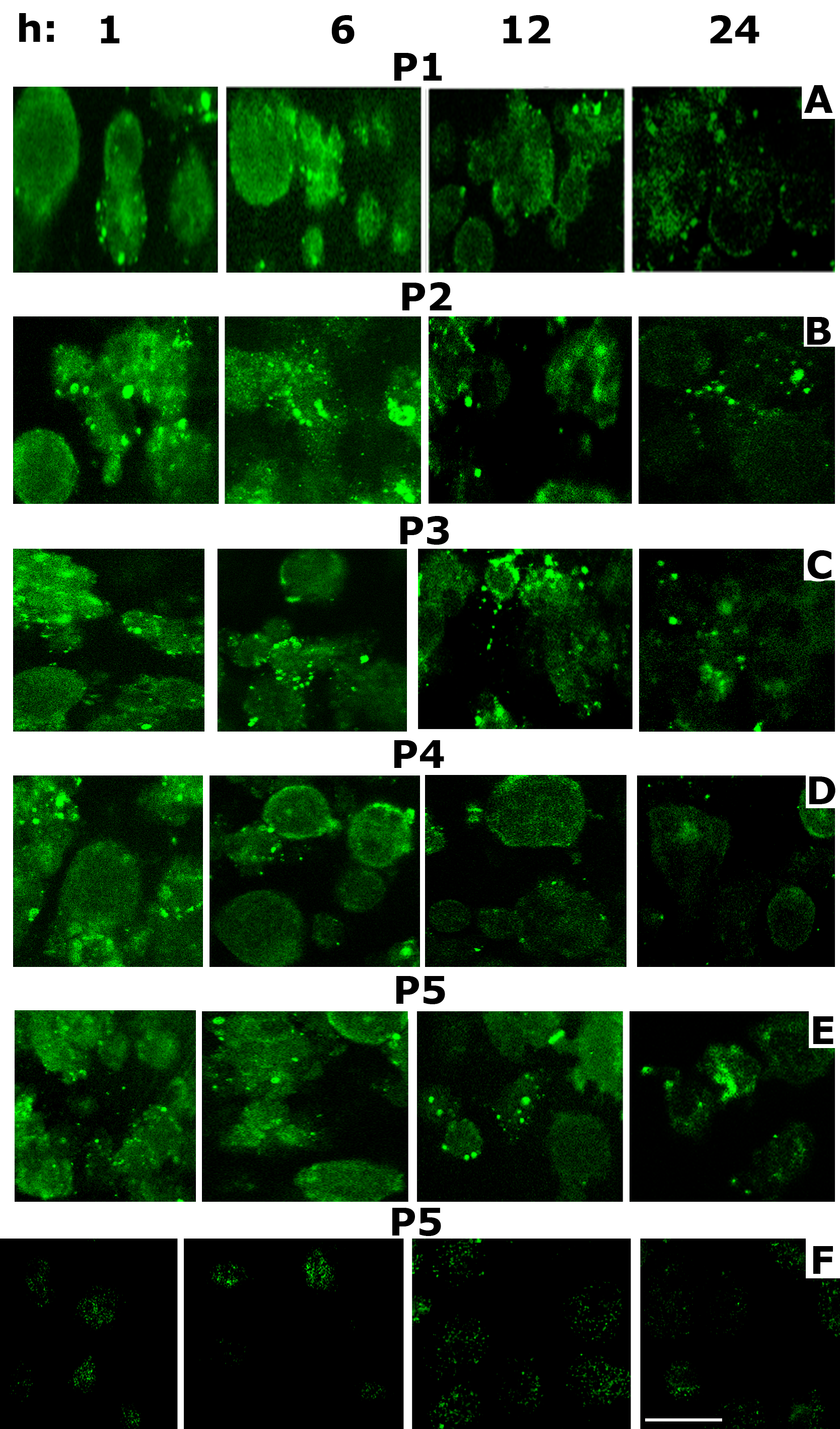

Supplement: S1 Fig — Confocal microscopy analysis of osteoclasts immunostained with a TAT- antibody (A-E) and a non-immune serum (F) is shown. TAT-stained osteoclasts at different times (1, 6, 12, and 24h) after transduction with indicated TAT-fused peptides are shown. Scale bar-150 μm. (TIF) [file pone.0204209.s001.tif]

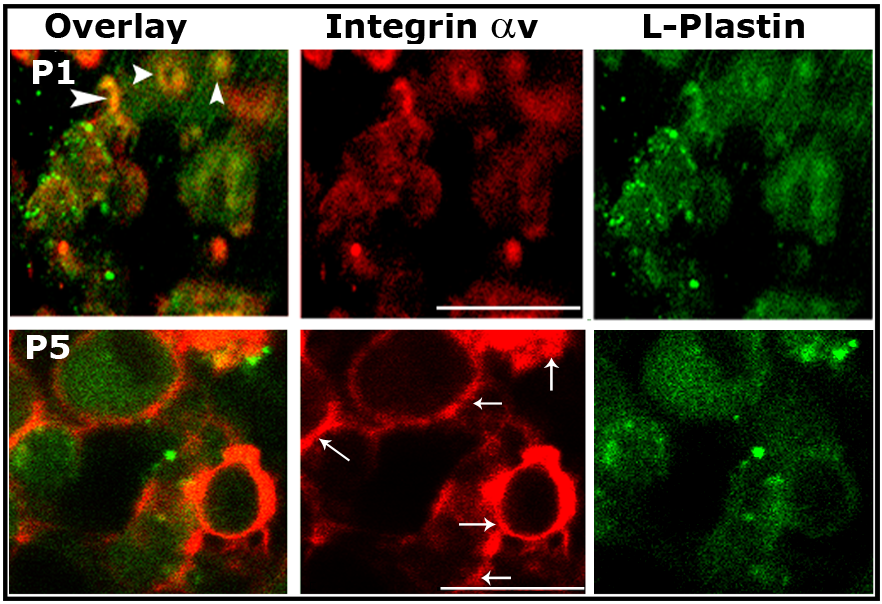

Supplement: S2 Fig — Osteoclasts transduced with P1 and P5 (scrambled) peptides for 10h were immunostained with an LPL (green) and Integrin αv (red) antibody. Sealing rings are indicated by arrows (P5; red panel). Arrowheads point to NSZs developing into mature sealing rings which demonstrate colocalization (yellow) of LPL (green) and integrin αv (red) (P1; overlay panel). Scale bar: 150μm. (TIF) [file pone.0204209.s002.tif]

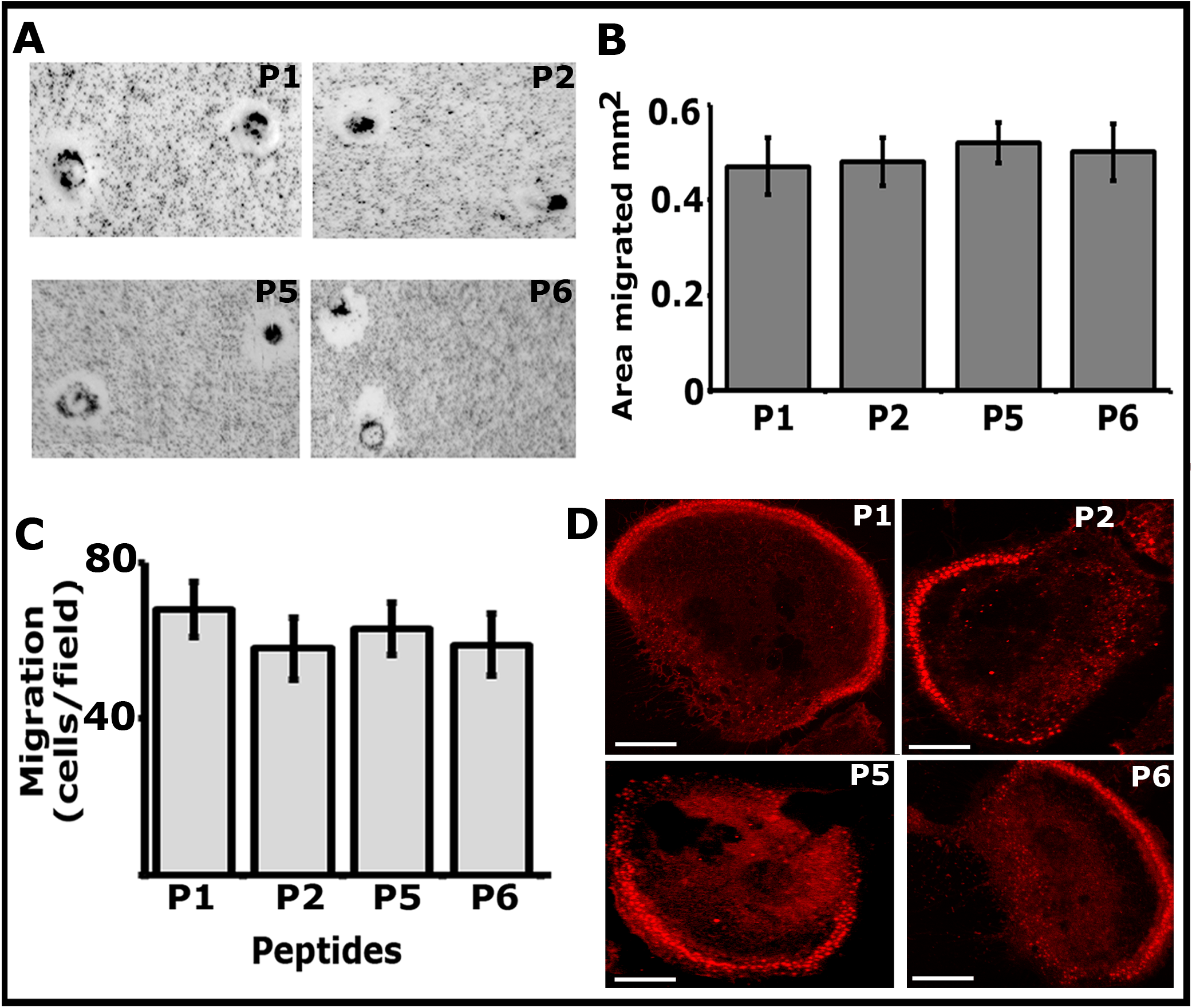

Supplement: S3 Fig — Motility (A-C) and podosome organization (D) was assessed in osteoclasts treated with peptides (P1, P2, P5, and P6). (A-C) Phagokinesis (A and B) and transwell migration (C) assays. The data in B are mean ± SD of 20–30 cell tracks (clear areas) represented as area migrated in mm2. The data in C are mean ± SD of migrated cells and provided as cells/filed. Images were taken in a phase contrast microscopy with a 10X objective (magnification X100). (D) Confocal microscopy analysis of rhodamine-phalloidin stained cells. None of the indicated peptides has any effect on osteoclast migration (A-C) or podosome organization (D). Podosome organization is seen at the periphery of osteoclasts. These results represent one of the three experiments performed with the similar results. Scale bar: 50μm. (TIF) [file pone.0204209.s003.tif]
